# Intraspecies Competition in Serratia marcescens Is Mediated by Type VI-Secreted Rhs Effectors and a Conserved Effector-Associated Accessory Protein

**DOI:** 10.1128/JB.00199-15

**Published:** 2015-06-19

**Authors:** Juliana Alcoforado Diniz, Sarah J. Coulthurst

**Affiliations:** Division of Molecular Microbiology, College of Life Sciences, University of Dundee, Dundee, United Kingdom

## Abstract

The type VI secretion system (T6SS) is widespread in Gram-negative bacteria and can deliver toxic effector proteins into eukaryotic cells or competitor bacteria. Antibacterial T6SSs are increasingly recognized as key mediators of interbacterial competition and may contribute to the outcome of many polymicrobial infections. Multiple antibacterial effectors can be delivered by these systems, with diverse activities against target cells and distinct modes of secretion. Polymorphic toxins containing Rhs repeat domains represent a recently identified and as-yet poorly characterized class of T6SS-dependent effectors. Previous work had revealed that the potent antibacterial T6SS of the opportunistic pathogen Serratia marcescens promotes intraspecies as well as interspecies competition (S. L. Murdoch, K. Trunk, G. English, M. J. Fritsch, E. Pourkarimi, and S. J. Coulthurst, J Bacteriol 193:6057–6069, 2011, http://dx.doi.org/10.1128/JB.05671-11). In this study, two new Rhs family antibacterial effectors delivered by this T6SS have been identified. One of these was shown to act as a DNase toxin, while the other contains a novel, cytoplasmic-acting toxin domain. Importantly, using S. marcescens, it has been demonstrated for the first time that Rhs proteins, rather than other T6SS-secreted effectors, can be the primary determinant of intraspecies competition. Furthermore, a new family of accessory proteins associated with T6SS effectors has been identified, exemplified by S. marcescens EagR1, which is specifically required for deployment of its associated Rhs effector. Together, these findings provide new insight into how bacteria can use the T6SS to deploy Rhs-family effectors and mediate different types of interbacterial interactions.

**IMPORTANCE** Infectious diseases caused by bacterial pathogens represent a continuing threat to health and economic prosperity. To counter this threat, we must understand how such organisms survive and prosper. The type VI secretion system is a weapon that many pathogens deploy to compete against rival bacterial cells by injecting multiple antibacterial toxins into them. The ability to compete is vital considering that bacteria generally live in mixed communities. We aimed to identify new toxins and understand their deployment and role in interbacterial competition. We describe two new type VI secretion system-delivered toxins of the Rhs class, demonstrate that this class can play a primary role in competition between closely related bacteria, and identify a new accessory factor needed for their delivery.

## INTRODUCTION

Bacteria utilize protein secretion systems to interact with and manipulate eukaryotic host cells, the abiotic environment, and other bacterial cells. Secretion systems, and the diverse proteins they secrete, represent critical determinants of competitive fitness and pathogenic potential. The type VI secretion system (T6SS) is widespread among Gram-negative bacteria and exhibits an intriguing versatility, being able to target effector proteins into both eukaryotic cells and competitor bacterial cells. While T6SS-mediated antieukaryotic activity can play an important role in virulence, for example, in Vibrio cholerae and Burkholderia species, antibacterial T6SSs can be key determinants of competitive fitness and are likely to shape the outcome of diverse polymicrobial infections (1–3). Indeed, it now appears likely that the primary function of the T6SS is to target bacterial cells, and many bacterial species have been reported to use antibacterial T6SSs against competitors, including Pseudomonas aeruginosa, V. cholerae, Serratia marcescens, and enteroaggregative Escherichia coli (4–7).

T6SSs are encoded by large gene clusters, which code for 13 essential core components together with various accessory, regulatory, and secreted proteins. The core components, named TssA-M, form a complex *trans*-envelope machinery which directly injects secreted effector proteins from the bacterial cytoplasm into a target cell. Current models (8, 9) suggest a bacteriophage-like mode of action for the T6SS, whereby contraction of a cytoplasmic TssBC sheath, anchored in a basal complex, propels a puncturing structure out from the secreting cell into the target cell. This extracellular puncturing structure is made up of Hcp (TssD), a small protein forming hexameric rings thought to stack into a phage tail tube-like structure, and VgrG (TssI). VgrG is structurally very similar to phage tail spike proteins and forms a trimer believed to sit on the distal end of the Hcp tube in order to mediate target cell puncturing via a C-terminal β-helical spike (10, 11). Small proline-alanine-alanine-arginine repeat proteins (PAAR proteins), or PAAR domains within larger proteins, interact with VgrG to form a final sharp tip on the spike (12).

Diverse effector proteins secreted by the T6SS have been identified recently, the majority of which are antibacterial toxins, and it is clear that individual systems can secrete multiple distinct effector proteins (2, 3). These include peptidoglycan amidase and peptidoglycan glycoside hydrolase effectors (targeting the bacterial cell wall), phospholipase effectors (targeting the cell membrane), and several nuclease enzymes (2, 3, 13). Each antibacterial effector is encoded by a gene adjacent to a gene encoding a specific, cognate immunity protein. These immunity proteins provide protection for the producing cell against its own effectors and against incoming effectors from neighboring sibling cells. T6SS-secreted effectors can be broadly divided into two classes (2). Many are standalone cargo effectors which are simply translocated by the system and are recruited through noncovalent interactions with Hcp, VgrG, or other components. Some, however, are specialized effectors in which an effector domain is covalently attached to a component of the extracellular puncturing device. The latter class is exemplified by the evolved VgrG proteins which have extra effector domains attached to their C termini, for example, the actin cross-linking domain of VgrG-1 (antieukaryotic) and peptidoglycan hydrolase domain of VgrG-3 (antibacterial) in V. cholerae (14, 15). Recent findings suggest that the fusion of effector domains to PAAR domains to enable their T6SS-dependent translocation is an alternative strategy that could be widespread, particularly within a large group of the so-called Rhs (for recombination hot spot) proteins (12, 16, 17). However, the roles and mechanisms of deployment of such effectors are not yet well defined. Rhs proteins are large polymorphic toxins with N-terminal regions defining their mode of secretion or attachment to the producing cell, central regions containing Rhs (or YD) repeat sequences, and highly variable C-terminal toxin domains (18, 19). Such polymorphic toxins normally are associated with specific immunity proteins encoded by genes immediately downstream (17, 19).

Serratia marcescens is an opportunistic pathogen capable of causing a range of infections; some strains were isolated as potent insect pathogens, while others came to attention as antibiotic-resistant clinical isolates causing problematic nosocomial infections (20). We previously identified a single antibacterial T6SS in S. marcescens Db10 which exerts potent killing activity against competitor bacteria, including closely related strains (7). A proteomic study identified six distinct cargo effectors, Ssp1-Ssp6, secreted by this system, with further studies demonstrating that Ssp1 and Ssp2 are related but not identical peptidoglycan amidase (Tae4) effectors which are neutralized by two unrelated immunity proteins, Rap1a and Rap2a (Tai4a and Tai4), respectively (21–23). Intriguingly, the proteomic study also revealed T6SS-dependent secretion of a PAAR-containing Rhs protein (22). This coincided with the report that RhsA and RhsB of Dickeya dadantii mediate growth inhibition of neighboring cells, with RhsB shown to require VgrG proteins for inhibition (16). This prompted us to consider the contribution of specialized effectors to the antibacterial activity of the S. marcescens Db10 T6SS. The genome of this organism does not encode any evolved VgrG or evolved Hcp proteins; however, in addition to the PAAR-containing Rhs protein seen in our earlier proteomic study, we further noted the presence of a gene encoding a second such protein. Therefore, we focused on these putative Rhs-family effectors. Here, we show that both of these Rhs proteins are T6SS-dependent antibacterial toxins and that Rhs proteins, rather than cargo effectors, appear to be the primary determinants of intraspecies competition between S. marcescens Db10 and closely related organisms. One Rhs effector has DNase activity, whereas the other represents a novel, cytoplasmic-acting toxin. Additionally, we describe a conserved accessory protein, EagR1, specifically required for T6SS-dependent deployment of the adjacently encoded Rhs effector.

## MATERIALS AND METHODS

### Bacterial strains, plasmids, and culture conditions.

Strains and plasmids used in this study are described in Table 1. Mutants constructed in S. marcescens Db10 were in-frame deletion mutants generated by allelic exchange using the pKNG101 suicide vector, and streptomycin-resistant derivatives were generated by phage ϕIF3-mediated transduction, both as described previously (7, 21). Plasmids for arabinose-inducible gene expression were derived from pBAD18-Kan (35) and for the DNase assay from pET15b (Novagen). Full details of plasmid construction and primer sequences are given in Table S1 in the supplemental material. S. marcescens was grown at 30°C in LB and Escherichia coli at 37°C in LB or M9 minimal medium (21). M9 contained 0.5% glycerol plus, as required, arabinose (routinely 0.02% or as stated) or glucose (routinely 0.5% or as stated). Kanamycin (Km) or streptomycin (Sm) was included in media at 100 μg/ml as required.

**TABLE 1:** Bacterial strains and plasmids used in this study

Name	Description	Source or reference
Strains		
S. marcescens		
Db10	Wild type	41
SJC3	Db10 Δ*tssH* (ΔSMDB11_2274)	7
SJC11	Db10 Δ*tssE* (ΔSMDB11_2271)	7
MJF15	Db10 Δ*rhs1* (ΔSMDB11_2278)	This study
JAD13	Db10 Δ*rhs2* (ΔSMDB11_1610)	This study
JAD14	Db10 Δ*rhs1* Δ*rhs2* (ΔSMDB11_2278 ΔSMDB11_1610)	This study
JAD03	Db10 Δ*rhsI1* Δ*tssH* (ΔSMDB11_2278A ΔSMDB11_2274)	This study
JAD16	Db10 Δ*rhs2* Δ*rhsI2* (ΔSMDB11_1610 ΔSMDB11_1611)	This study
JAD12	Db10 Δ*eagR1* (ΔSMDB11_2277)	This study
JAD09	Sm^r^ derivative of JAD03 (Δ*rhsI1* Δ*tssH*)	This study
JAD17	Sm^r^ derivative of JAD16 (Δ*rhs2* Δ*rhsI2*)	This study
JAD06	Sm^r^ derivative of JAD01 (Δ*ssp4* Δ*sip4*)	22
SJC17	Sm^r^ derivative of S. marcescens ATCC 274	7
SM39	Wild-type strain (intrinsically Sm^r^)	N. Gotoh
Enterobacter cloacae ATCC 13047	Wild-type strain (intrinsically Sm^r^)	ATCC
Pseudomonas fluorescens KT02	Sm^r^ derivative of P. fluorescens 55	7
Escherichia coli		
MC4100	Model K-12 strain; Sm^r^ (*rpsL150*)	42
MG1655	Wild type (model K-12 strain)	43
BL21(DE3) pLysS	Protein overexpression host carrying the λDE3 lysogen, allowing IPTG-inducible expression of T7 RNA polymerase, and pLysS, inhibiting basal levels of T7 polymerase in the absence of induction	21
CC118λ*pir*	Cloning host and donor strain for pKNG101-derived allelic exchange plasmids (λ*pir*)	44
HH26 pNJ5000	Mobilizing strain for conjugal transfer	45
Plasmids		
pBAD18-Kan	Arabinose-inducible expression vector (Km^r^); gene of interest is cloned downstream of the P*_ara_* promoter	35
pET15b-TEV	Protein overexpression vector for T7 polymerase-dependent expression of recombinant proteins, derived from pET15b	21
pKNG101	Suicide vector for marker exchange (Sm^r^ *sacBR mobRK2 oriR6K*)	46
pSC635	Coding sequence for Rhs1-CT (SMDB11_2278; amino acids 1333 to 1473) in pBAD18-Kan	This study
pSC636	Coding sequence for RhsI1 (SMDB11_2278A) in pBAD18-Kan	This study
pSC637	Coding sequences for Rhs1-CT plus RhsI1 in pBAD18-Kan	This study
pSC640	Coding sequence for RhsI1 with a C-terminal HA tag in pBAD18-Kan	This study
pSC643	Coding sequence for full-length Rhs1 (SMDB11_2278) in pBAD18-Kan	This study
pSC658	Coding sequence for EagR1 (SMDB11_2277) in pBAD18-Kan	This study
pSC672	Coding sequence for RhsI2 (SMDB11_1611) in pBAD18-Kan	This study
pSC673	Coding sequences for RhsI1 and RhsI2 in pBAD18-Kan	This study
pSC674	Coding sequence for Rhs2-CT (SMDB11_1610; amino acids 1290 to 1430) in pET15b-TEV (no affinity tag fused to Rhs2-CT)	This study
pSC675	Coding sequences for Rhs2-CT plus RhsI2 in pET15b-TEV (no affinity tag fused to either protein)	This study
pSC619	pKNG101-derived allelic exchange plasmid for the generation of chromosomal in-frame Δ*SMDB11_2278A* (Δ*rhsI1*) deletion	This study
pSC649	pKNG101-derived allelic exchange plasmid for the generation of chromosomal in-frame Δ*SMDB11_2277* (Δ*eagR1*) deletion	This study
pSC650	pKNG101-derived allelic exchange plasmid for the generation of chromosomal in-frame Δ*SMDB11_1610* (Δ*rhs2*) deletion	This study
pSC664	pKNG101-derived allelic exchange plasmid for the generation of chromosomal in-frame Δ*SMDB11_1610-1611* (Δ*rhs2* Δ*rhsI2*) deletion	This study
pSC827	pKNG101-derived allelic exchange plasmid for the generation of chromosomal in-frame Δ*SMDB11_2278* (Δ*rhs1*) deletion	This study

### Coculture assays for T6SS-mediated antibacterial activity.

Coculture assays were based on the assay described previously (7). In brief, attacker strain and target strain were mixed at an initial attacker/target ratio of either 1:1 (target strain Escherichia coli MC4100, S. marcescens SM39, or a mutant of S. marcescens Db10) or 5:1 (target strain P. fluorescens, Enterobacter cloacae, or S. marcescens ATCC 274) and cocultured on solid LB for 4 h at 30°C (except for 7.5 h for S. marcescens Db10 target strains and 37°C when Escherichia coli was used as the target). Following coculture, the surviving target cells were enumerated by serial dilution and viable counts on streptomycin-containing media. The target strain was always the streptomycin-resistant version of the organism or mutant in question (Table 1). The means from independent biological replicates is reported, normally four and always at least three replicates.

### Immunodetection of secreted proteins.

Anti-Hcp1, anti-Ssp1, and anti-Ssp2 immunoblots were performed as described previously (7, 21), except that cultures were grown for 5 h.

### Toxicity assays.

To observe the impact of heterologous expression of proteins of interest from pBAD18-Kan-based plasmids, fresh transformants of Escherichia coli MG1655 were resuspended in media, adjusted to an optical density at 600 nm (OD_600_) of 1, and serially diluted, and 5 μl was spotted onto LB or M9 minimal medium agar containing either 0.2% d-glucose or l-arabinose. To detect nuclease activity, fresh transformants of BL21(DE3) pLysS were grown to an OD_600_ of 0.8 in LB at 37°C, induced by addition of isopropyl-β-d-thiogalactopyranoside (IPTG) to a final concentration of 1 mM, and grown for 1 h at 19°C. Plasmid DNA was prepared from each culture and separated by agarose gel electrophoresis.

### Localization of RhsI-HA protein.

Following growth of cultures for 5 h in LB containing kanamycin and 0.1% arabinose, cells were fractionated using a cold osmotic shock procedure as described previously (36), except that Na_2_CO_3_ was added prior to sonication. Anti-RNA polymerase β subunit (Neoclone, USA) was used at 1:20,000 and anti-maltose binding protein (NEB) at 1:10,000, both with peroxidase-conjugated anti-mouse secondary antibody (Roche) at 1:10,000. Horseradish peroxidase (HRP)-conjugated antihemagglutinin (anti-HA) (Roche) was used at 1:10,000. Anti-TssJ (37) was used at 1:4,000, with peroxidase-conjugated anti-rabbit secondary antibody (Thermo Fisher Scientific) at 1:10,000.

### *In silico* analyses.

Bioinformatic analysis of Rhs, RhsI, and SMDB11_2277-like proteins and T6SS gene clusters in strains of S. marcescens utilized the sequence databases and BLAST servers at the NCBI (www.ncbi.nlm.nih.gov) and the Pfam database (http://pfam.xfam.org/). The complete genome sequence of S. marcescens Db11 was used for all analyses of Db10, since Db11 is a spontaneous Sm^r^ derivative of Db10 and the strains differ in only one nucleotide position (20). To construct the DUF1795/PF08786 family alignment, a set of homologues was retrieved from UniProtKB (http://www.uniprot.org/), based on the members of the Pfam seed alignment for this family augmented with other homologues of interest. The multiple-sequence alignment was generated using Clustal Omega (38), and Jalview (39) was used to visualize the alignment and to calculate the resulting tree (using neighbor-joining construction and BLOSUM62 distance measure). A model of the structure of SMDB11_2277 (EagR1) was generated using Phyre2 (40) based on the structure of PA0094 (PDB entry 1TU1).

## RESULTS

### S. marcescens Db10 encodes two putative T6SS-dependent Rhs/RhsI pairs, and these are not conserved in other strains of S. marcescens.

**FIG 1:**
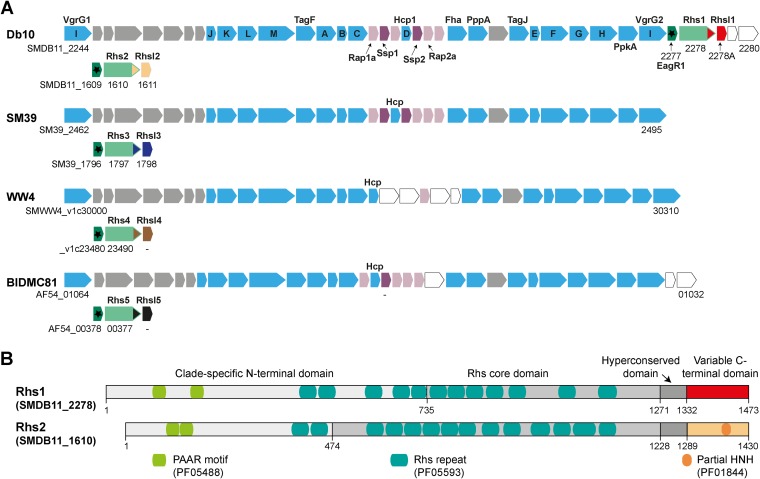
Type VI secretion system-associated Rhs proteins in Serratia marcescens. (A) Comparison of T6SS gene clusters and distant loci encoding PAAR domain-containing Rhs proteins between S. marcescens Db10 and three other strains of S. marcescens: SM39, WW4, and BIDMC81. Conserved T6SS components are shown in blue, with core components TssA-M indicated by single letters and others indicated by common names (VgrG and Hcp are core components TssI and TssD). Tae4 family effectors are shown in purple, Tai4/4a family immunity proteins are in pink, uncharacterized genes conserved in S. marcescens are in gray, and strain-specific genes are white. Genes encoding Rhs proteins and EagR accessory proteins (DUF1795; asterisk) are shown in green; distinct C-terminal domains and putative cognate RhsI proteins are indicated with different colors. Sequence data were obtained from NCBI databases, and genomic identifiers are given for selected genes (a dash indicates a nonannotated open reading frame manually identified as likely encoding an RhsI or Tae4 protein). (B) The domain organization of Rhs proteins of S. marcescens Db10. The Rhs domains are as defined in reference 18, and positions of PAAR motifs, Rhs repeats, and a partial HNH endonuclease domain are indicated. Amino acid numbering is given below each protein.

As mentioned above, we had previously observed T6SS-dependent secretion of SMDB11_2278, a PAAR domain-containing Rhs family protein encoded by a gene within the main T6SS gene cluster of S. marcescens Db10 (Fig. 1A). Further examination of the genome of S. marcescens Db10 revealed the presence of a second such Rhs protein, encoded elsewhere in the genome by SMDB11_1610. Reexamination of our proteomic data from the earlier study (22) indicated that SMDB11_1610 also can be secreted in a T6SS-dependent manner: although missing our stringent quality criteria, since only one peptide was detected, the presence of this peptide in the secreted fraction was strictly T6SS dependent (data not shown). Thus, we named SMDB11_2278 Rhs1 and SMDB11_1610 Rhs2. Examination of the protein sequences of Rhs1 and Rhs2 confirmed the domain organization expected for a T6SS-associated Rhs protein (Fig. 1B). PAAR motifs are present in the N-terminal domain of each protein, followed by a central region containing multiple Rhs repeats and including an Rhs core domain which terminates in the so-called hyperconserved domain (18, 19). Finally, each possesses a distinct C-terminal domain (CT), unrelated between the two proteins, which is predicted to be a toxin or effector domain. The C-terminal domain of Rhs2 (Rhs2-CT) contains a partial HNH endonuclease domain (see Fig. S1 in the supplemental material), suggesting DNase activity, whereas the function of the C-terminal domain of Rhs1 (Rhs1-CT) is not readily apparent from sequence analysis. It is noteworthy that Rhs1 and Rhs2 belong to distinct clades of Rhs-family proteins, clade III and clade II, respectively (18).

Immediately downstream of each Rhs protein is a small open reading frame predicted to encode the immunity protein providing self-protection against the cognate Rhs-CT. These were named RhsI1 (SMDB11_2278A) and RhsI2 (SMDB11_1611). Consistent with the distinct Rhs-CTs, these proteins are unrelated to each other; they also have no predicted domains or homologues of known function (data not shown).

Examination of the genome sequences of three other strains of S. marcescens revealed the presence of T6SS gene clusters closely related to those of S. marcescens Db10 but with the notable absence of Rhs1 (Fig. 1A). However, each strain does encode an Rhs family protein at a distant genomic location. These all are closely related to Rhs2 of S. marcescens Db10, with the exception of their C-terminal domains, which are unique to each strain. Correspondingly, each has a distinct, unrelated small open reading frame immediately downstream, predicted to encode the cognate RhsI immunity protein. These pairs of proteins were named Rhs3-5 and RhsI3-5 (Fig. 1A). Only Rhs5-CT contains a recognizable conserved domain of unknown function, DUF3990 (pfam13151).

### Rhs1 and Rhs2 are specifically required for T6SS-mediated killing of strains lacking the immunity proteins RhsI1 and RhsI2.

**FIG 2:**
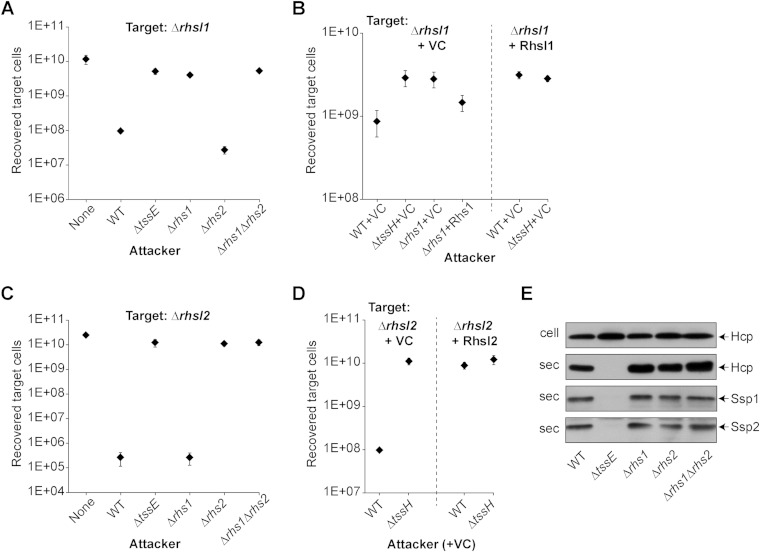
Mutants of Serratia marcescens lacking the immunity determinant RhsI1 or RhsI2 are sensitive to the action of type VI secretion system-delivered toxin Rhs1 or Rhs2, respectively. (A) Number of target cells recovered following coculture of a target strain lacking *rhsI1* (S. marcescens Db10 Δ*rhsI1* Δ*tssH* mutant) with wild-type (WT) or mutant (Δ*tssE*, Δ*rhs1*, Δ*rhs2*, and Δ*rhs1* Δ*rhs2*) strains of S. marcescens Db10 as the attacker. None indicates coculture of the target with sterile medium alone, and the Δ*tssE* mutant has a nonfunctional T6SS. (B) Recovery of the *rhsI1* mutant carrying either a vector control plasmid (Δ*rhsI1*+VC; the Δ*rhsI1* Δ*tssH* mutant with pBAD18-Kan) or complementing plasmid expressing *rhsI1* in* trans* (Δ*rhsI1*+RhsI1; Δ*rhsI1* Δ*tssH* mutant with pSC636) as the target strain following coculture with attacker strains carrying either a vector control plasmid (VC; pBAD18-Kan) or a complementing plasmid expressing *rhs1* in* trans* (+Rhs1; pSC643) as indicated. (C) Recovery of a target strain lacking *rhsI2* (Db10 Δ*rhs2* Δ*rhsI2* mutant) following coculture with WT or mutant (Δ*tssE*, Δ*rhs1*, Δ*rhs2*, and Δ*rhs1* Δ*rhs2*) strains of S. marcescens Db10 as the attacker. (D) Recovery of the *rhsI2* mutant carrying either a vector control plasmid (Δ*rhsI2*+VC; Δ*rhs2* Δ*rhsI2* mutant with pBAD18-Kan) or complementing plasmid expressing *rhsI2* in* trans* (Δ*rhsI2*+RhsI2; Δ*rhs2* Δ*rhsI2* mutant with pSC672) as the target strain following coculture with WT or Δ*tssH* attacker strains also carrying the vector control plasmid (VC; pBAD18-Kan). (A to D) Points show means ± standard errors of the means (SEM) (*n* ≥ 3). (E) Immunoblot detection of Hcp, Ssp1, and Ssp2 in cellular (cell) and secreted (sec) fractions of WT or mutant strains of S. marcescens Db10.

To confirm the putative functions of Rhs1 and RhsI1 as T6SS-delivered effector and cognate immunity proteins, respectively, we attempted to demonstrate T6SS- and Rhs1-dependent antibacterial activity of wild-type S. marcescens Db10 against a mutant lacking *rhsI1*. We were unable to construct the Δ*rhsI1* mutant in a wild-type genetic background, consistent with self-toxicity of such a strain. However, a Δ*rhsI1* Δ*tssH* double mutant, where TssH is an essential component of the T6SS (7), was readily generated and fully fit. When used as the target in a coculture assay, this mutant lacking RhsI1 was indeed susceptible to T6SS-mediated inhibition by the wild type, showing a 40-fold drop in its recovery in the presence of the wild type compared to that with a T6SS mutant attacker (Fig. 2A). Further, this antibacterial activity was entirely and specifically dependent on the presence of Rhs1 in the attacker strain, being eliminated from a Δ*rhs1* mutant but not a Δ*rhs2* mutant. In* trans* expression of Rhs1 was able to partially restore this antibacterial activity to the Δ*rhs1* mutant, and complementation of the Δ*rhsI1* mutant by expression of *rhsI1* eliminated its sensitivity to the wild type (Fig. 2B). The same approach then was used to demonstrate an effector immunity function for Rhs2-RhsI2. A mutant lacking RhsI2, specifically a Δ*rhs2* Δ*rhsI2* mutant to avoid self-toxicity, was constructed. This strain was highly susceptible to T6SS-mediated antibacterial activity of the wild-type strain, showing a 50,000-fold drop in recovery (Fig. 2C). This activity again was dependent on the presence of the cognate Rhs protein in the attacker, being eliminated in a Δ*rhs2* (but not Δ*rhs1*) mutant. Complementation of this Δ*rhsI2* mutant by expression of RhsI2 in *trans* restored its resistance to the wild-type strain (Fig. 2D). Additionally, to confirm that the loss of antibacterial activity in the Rhs mutants was not due to a loss of T6SS functionality, T6SS-dependent secretion of Hcp1 and the two effectors, Ssp1 and Ssp2, to the extracellular medium was shown to be unaffected in the single and double *rhs* mutants (Fig. 2E). This was not unexpected, since a nonspecialized PAAR protein is also present in this organism which should support assembly of a functional T6SS (12).

### The Rhs2 C-terminal domain is a DNase toxin.

**FIG 3:**
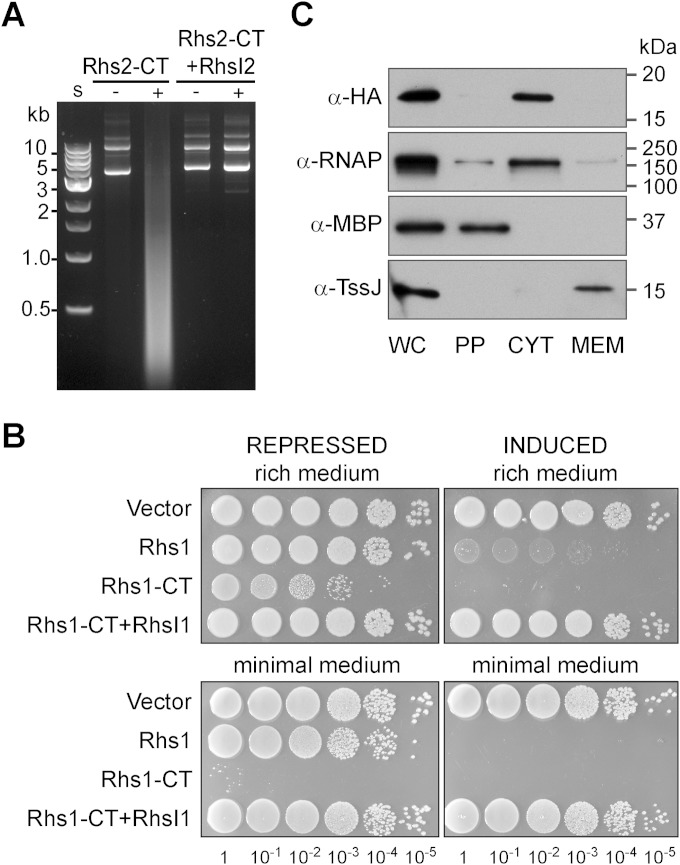
C-terminal domain of Rhs2 has DNase activity, and the C-terminal domain of Rhs1 is a cytoplasmic-acting antibacterial toxin. (A) Degradation of plasmid DNA is observed on expression of Rhs2-CT in Escherichia coli. Plasmid DNA recovered from Escherichia coli BL21(DE3) pLysS carrying plasmids expressing the C-terminal domain of Rhs2 (Rhs2-CT; pSC674) or Rhs2-CT with RhsI2 (Rhs-CT+RhsI2; pSC675), with (+) or without (−) IPTG induction. S, size standards. (B) Heterologous expression of Rhs1-CT in Escherichia coli is toxic and alleviated on coexpression of RhsI1. Serial dilutions of Escherichia coli MG1655 carrying the empty vector (VC; pBAD18-Kan) or plasmids expressing full-length Rhs1 (Rhs1; pSC643), the C-terminal domain of Rhs1 (Rhs1-CT; pSC635), or Rhs1-CT with RhsI1 (Rhs-CT+RhsI1; pSC637) were spotted onto rich medium (LB) or M9 minimal medium. Gene expression was repressed or induced by the inclusion of 0.2% d-glucose or l-arabinose, respectively, in the media. (C) Subcellular localization of RhsI1. Cells of S. marcescens Db10 expressing an RhsI1-HA fusion protein (from pSC640) were subjected to fractionation and immunoblotting using antibodies against the HA tag, RNAP (RNA polymerase β subunit; cytoplasmic control), MBP (maltose binding protein; periplasmic control), or TssJ (membrane control). WC, whole cell; PP, periplasm; CYT, cytoplasm; MEM, total membrane.

We predicted that the C-terminal domain of Rhs2 could have DNase activity, since it shows similarity to HNH endonuclease domains (see Fig. S1 in the supplemental material) and shares 31% identity with the CT of RhsB of D. dadantii, which exhibits DNase activity (16). Induction of Rhs2-CT expression in Escherichia coli, using a tightly regulated T7 polymerase-based system, resulted in degradation of plasmid DNA (Fig. 3A). When RhsI2 was coexpressed, no degradation occurred, confirming a direct immunity function for this protein.

### The Rhs1 C-terminal domain represents a novel antibacterial toxin.

Unlike Rhs2-CT, the activity of Rhs1-CT is not obvious from bioinformatic analysis. Therefore, we first demonstrated that Rhs1-CT does represent an antibacterial toxin by inducible heterologous expression in the cytoplasm of Escherichia coli. As shown in Fig. 3B, induction of Rhs1-CT prevented any growth of Escherichia coli on rich or minimal medium, with growth on minimal medium almost eliminated even under repressive conditions. Importantly, coexpression of RhsI1 was able to completely neutralize this toxic effect. Expression of full-length Rhs1 was also toxic, but less dramatically so than that of the isolated CT. This observation of potent cytotoxicity in the cytoplasm of Escherichia coli suggested a cytoplasmic site of action for the toxin. To support this contention, the subcellular localization of RhsI1 was determined using an Rhs1-HA fusion protein, which retained the ability to restore resistance in an Δ*rhs1* mutant similar to that of the wild-type allele (data not shown). Fractionation of cells of S. marcescens Db10 expressing RhsI1-HA followed by immunoblotting revealed that RhsI is localized in the cytoplasm (Fig. 3C). Rhs1-CT did not show any DNase activity (data not shown) and did not appear to be as exquisitely toxic as Rhs2-CT. It was impossible to clone Rhs2-CT in the absence of RhsI2 in the expression system used for RhsI1-CT (Fig. 3B), and the degree of killing of the respective nonimmune mutant is even greater for Rhs2 than Rhs1 (Fig. 2). Hence, Rhs1 represents a novel antibacterial toxin, active in the cytoplasm of target cells and neutralized by RhsI1.

### Rhs proteins are particularly important in intraspecies competition.

**FIG 4:**
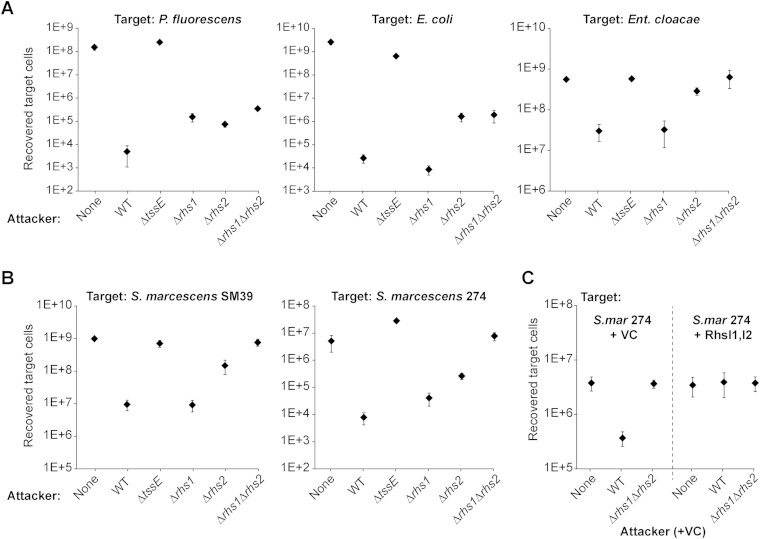
Contribution of Rhs proteins to type VI secretion system-mediated inter- and intraspecies antibacterial activity of Serratia marcescens Db10. (A) Recovery of target organisms P. fluorescens 55, Escherichia coli MC4100, and Enterobacter cloacae ATCC 13047 following coculture with wild-type (WT) or mutant (Δ*tssE*, Δ*rhs1*, Δ*rhs2*, and Δ*rhs1* Δ*rhs2*) strains of S. marcescens Db10 as the attacker. None indicates coculture of the target with sterile medium alone. Points show means ± SEM (*n* ≥ 3). (B) Recovery of target organisms S. marcescens SM39 and S. marcescens ATCC 274 following coculture with the strains of S. marcescens Db10 described for panel A. Points show means ± SEM (*n =* 4). (C) Recovery of S. marcescens ATCC 274 carrying the empty vector (VC; pBAD18-Kan) or a plasmid expressing RhsI1 and RhsI2 (RhsI1,I2; pSC673) following coculture with the WT or Δ*rhs1* Δ*rhs2* strain of Db10 carrying an empty vector. Points show means ± SEM (*n =* 4).

Having shown that Rhs1 and Rhs2 of S. marcescens Db10 are T6SS-delivered toxins, we asked what contribution they make toward T6SS-mediated antibacterial activity against competitor organisms. First, we used three non-Serratia species, Pseudomonas fluorescens, Escherichia coli, and Enterobacter cloacae, as targets (Fig. 4A). Against P. fluorescens, single Δ*rhs1* and Δ*rhs2* and double Δ*rhs1* Δ*rhs2* mutant attackers showed a modest decrease in antibacterial activity. Against Escherichia coli, the Δ*rhs1* mutant was not impaired, whereas the Δ*rhs2* and Δ*rhs1* Δ*rhs2* mutants again showed a modest decrease in antibacterial activity. However, against both target organisms, even the Δ*rhs1* Δ*rhs2* mutant retained considerable killing activity, implying that other effectors make an important contribution. In contrast, the Δ*rhs1* Δ*rhs2* mutant was no longer able to inhibit Enterobacter cloacae, behaving indistinguishably from a Δ*tssE* (T6SS mutant) attacker, with most of the effect attributable to Rhs2. Therefore, Rhs2 is the primary effector acting against this target organism. Enterobacter cloacae is closely related to S. marcescens and has a similar T6SS; therefore, we examined the contribution of Rhs to the ability of S. marcescens Db10 to compete with even more closely related targets, namely, other strains of S. marcescens (Fig. 4B). Against S. marcescens SM39, the Δ*rhs1* Δ*rhs2* mutant of Db10 was completely attenuated, with the single Δ*rhs2* mutant also severely impaired. Against S. marcescens ATCC 274, the double Δ*rhs1* Δ*rhs2* mutant retained negligible antibacterial activity, and this time both single *rhs* mutants showed reduced activity.

To distinguish the direct contribution of the Rhs toxin domains from any indirect impact of Rhs on the activity of other effectors, a plasmid expressing RhsI1 and RhsI2 was introduced into the S. marcescens ATCC 274 target strain. The resulting strain should be resistant to Rhs1-CT and Rhs2-CT but not any other effectors. If the antibacterial impact of the Rhs proteins against S. marcescens ATCC 274 is purely due to CTs, then there should be no difference in the recovery of the immune RhsI1^+^ RhsI2^+^ target strain between a wild-type and a Δ*rhs1* Δ*rhs2* mutant attacker. This is indeed the case, as shown in Fig. 4C. Altogether, the data in Fig. 4 are in accord with our observations that Rhs-CT/RhsI pairs are highly variable between strains of S. marcescens (Fig. 1A). Importantly, our findings demonstrate that Rhs proteins can be the primary effectors mediating competitiveness between closely related organisms.

### SMDB11_2277 (EagR1) is a conserved, essential accessory factor of Rhs1.

Encoded by a gene immediately upstream of that encoding Rhs1 is SMDB11_2277, a conserved protein of unknown function. Homologues of SMDB11_2277 are also encoded upstream of Rhs2-Rhs5 in S. marcescens (Fig. 1A) and upstream of T6SS-associated Rhs proteins in other organisms (Dda3937_01759 and Dda3937_02772 with RhsA and RhsB of D. dadantii; STM0290, also known as SciW [24], with Rhs in Salmonella enterica serovar Typhimurium; and ECL_01566 and ECL_03141 upstream of RhsA and RhsB in Enterobacter cloacae). In light of this and later results, we named SMDB11_2277 EagR1 (for effector-associated gene, with Rhs1). We hypothesized that EagR1 and its homologues are involved in the delivery or action of Rhs toxins.

**FIG 5:**
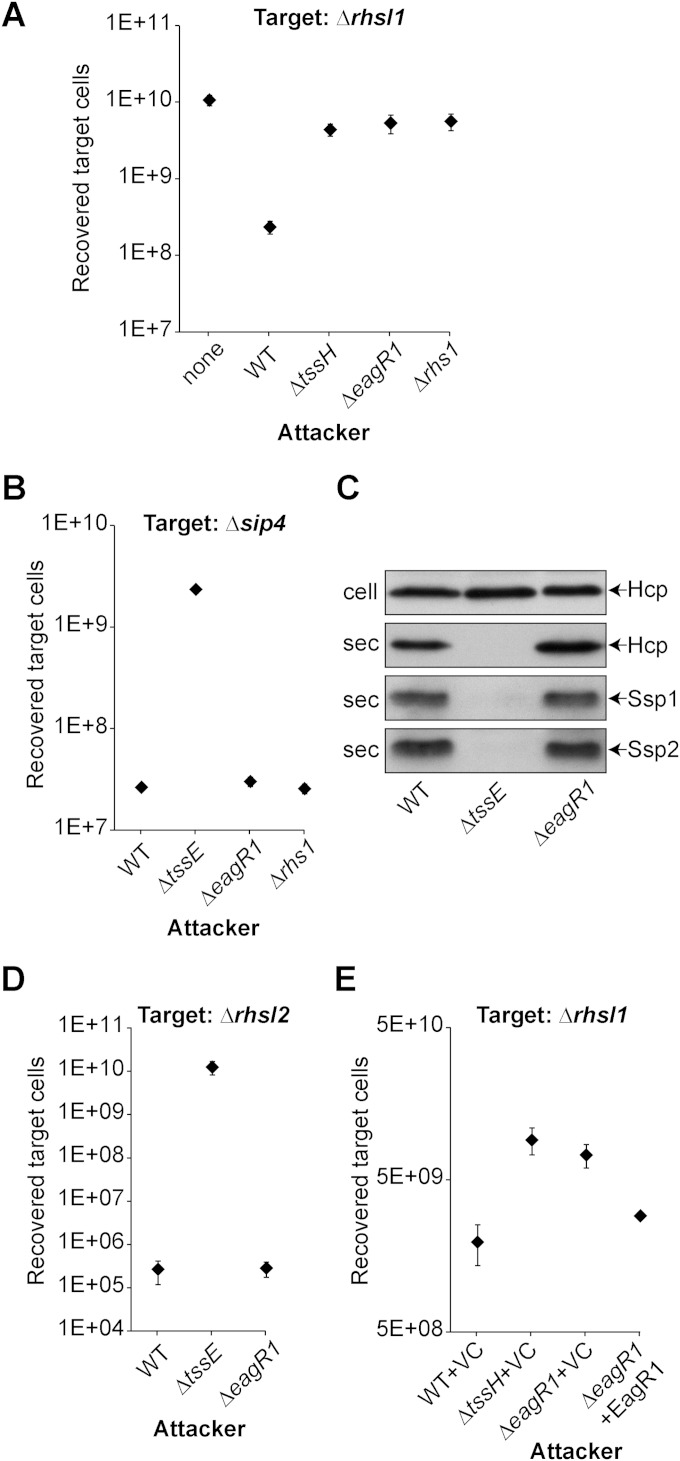
DUF1795 family protein EagR1 is specifically required for Rhs1-mediated antibacterial activity. (A and B) Recovery of target organism S. marcescens Db10 Δ*rhsI1* Δ*tssH* (Δ*rhsI1*) strain or S. marcescens Db10 Δ*ssp4* Δ*sip4* (Δ*sip4*) strain following coculture with wild-type (WT) or mutant (Δ*tssE*, Δ*eagR1*, and Δ*rhs1*) strains of S. marcescens Db10 as the attacker. The Δ*eagR1* mutant is an in-frame deletion mutant of SMDB11_2277. (C) Immunoblot detection of Hcp, Ssp1, and Ssp2 in cellular (cell) and secreted (sec) fractions of WT or mutant strains of S. marcescens Db10. (D) Recovery of target organism S. marcescens Db10 Δ*rhs2* Δ*rhsI2* (Δ*rhsI2*) strain following coculture with WT or mutant (Δ*tssE* and Δ*eagR1*) strains of S. marcescens Db10 as the attacker. This is part of the same experiment as that shown Fig. 2C, and the data for the control strains are repeated from that figure. (E) Recovery of the *rhsI1* mutant carrying a vector control plasmid (Δ*rhsI1*; Δ*rhsI1* Δ*tssH* mutant with pBAD18-Kan) as the target strain following coculture with attacker strains carrying either a vector control plasmid (VC; pBAD18-Kan) or complementing plasmid expressing SMDB11_2277 in *trans* (+2277; pSC658). Points show means ± SEM throughout (*n* ≥ 4).

To determine whether EagR1 is required for Rhs1-dependent antibacterial activity, we constructed a strain carrying an in-frame deletion in the cognate gene. This Δ*eagR1* mutant was unable to inhibit the Δ*rhsI1* mutant, the same phenotype as that of the Δ*rhs1* mutant (Fig. 5A). Additionally, again like the Δ*rhs1* mutant, the Δ*eagR1* mutant showed wild-type antibacterial activity against a Δ*sip4* target strain, which is susceptible to killing by the unrelated effector Ssp4 (22) (Fig. 5B). The latter result suggested that the Δ*eagR1* mutation did not cause a general impact on the function of the T6SS, further supported by the observation that secretion of Hcp1 and the effectors Ssp1 and Ssp2 was unaffected in the mutant (Fig. 5C). Additionally, we confirmed that EagR1 is not required for Rhs2-dependent killing of a Δ*rhsI2* mutant (Fig. 5D). Hence, the critical role of EagR1 is specific to Rhs1. Expression of EagR1 in *trans* in the Δ*eagR1* mutant was able to restore activity against the Δ*rhsI1* target (Fig. 5E). Finally, we confirmed that EagR1 is not in itself a toxin, since its expression in either the cytoplasm or periplasm of Escherichia coli had no impact on growth (data not shown). Hence, EagR1 is an essential, specific accessory factor for the Rhs1 effector toxin.

EagR1 and its Rhs-associated homologues are members of the DUF1795 (pfam08786) protein family. Multiple-sequence alignment of members of this family reveals regions of high conservation (see Fig. S2 in the supplemental material). The atomic structure of another member of this family, the PA0094 protein, encoded by a gene within the HSI-1 T6SS gene cluster of P. aeruginosa, has been deposited in the database. Therefore, we modeled the structure of EagR1 based upon this and mapped on the positions of these conserved residues (see Fig. S3). This analysis indicated that EagR1 should adopt a compact structure with a concave face, which may represent an interaction surface with Rhs1 or another binding partner. It is notable that the conserved residues all map to this concave side, whereas the back of the molecule shows only low conservation. This model provides a first glimpse of a conserved T6SS effector accessory protein.

## DISCUSSION

In this study, we have demonstrated that Rhs1 and Rhs2 of S. marcescens Db10 are new T6SS-dependent antibacterial effectors. Rhs2 is a DNase toxin, whereas Rhs1 carries a novel toxin domain. While this study was under way, RhsP1/Tse5 of P. aeruginosa and RhsA and RhsB of Enterobacter cloacae also were reported to be antibacterial toxins dependent on the T6SS and specific VgrG proteins for their deployment (25, 26), and IdrD has been implicated in T6SS-dependent self-recognition in Proteus mirabilis (27). However, the mode of toxicity of these other Rhs proteins has not yet been defined. Importantly, our findings reveal that Rhs effectors can play a critical role during intraspecies competition and in competition between closely related species. This is the first demonstration of Rhs-mediated interstrain competition and supports the prediction that different serovars of Salmonella deploy distinct Rhs toxins (28).

The observed importance of Rhs effectors during intraspecies competition is consistent with the high genetic variability associated with *rhs* loci, in S. marcescens and beyond (17, 18, 28). Interrogation of the genomic databases indicates that Rhs2 (clade II) is widespread across strains of S. marcescens, whereas Rhs1 (clade III) is confined to a small subset; nevertheless, for both types, distinct Rhs-CT/RhsI pairs are observed in different strains, including the four shown in Fig. 1. In general, it appears that Rhs-CT/RhsI pairs are readily acquired and exchanged by horizontal gene transfer via homologous recombination within the conserved core regions of Rhs (18, 28). Acquisition and deployment of a new Rhs-CT/RhsI pair might represent one way in which a new isolate of a particular species could arise through the enhanced ability of the recipient clone to outcompete the parental strain or a newly encountered competitor species. A genetic record of previous Rhs-CT/RhsI acquisition events can be seen in many enterobacterial genomes in the form of orphan or displaced Rhs-CT/RhsI genes located immediately 3′ of the intact Rhs/RhsI genes, including downstream of Rhs2 in S. marcescens Db10 (17, 18, 28). In fact, RhsI5 is 93% identical to the product of one of these genes downstream of Rhs2/RhsI2, suggesting that an ancestor of Db10 carried an intact *rhs5-rhsI5* pair. Indeed, it was reported recently that extended passage of S. enterica serovar Typhimurium yielded an evolved subpopulation able to inhibit parental cells by deployment of an orphan Rhs-CT following the fusion of this Rhs-CT with the main ancestral Rhs protein through a recombination event (28).

This study revealed the S. marcescens Db10 Rhs toxins to be potent weapons, conferring highly efficient killing of susceptible S. marcescens strains. However, against more distantly related target strains, such as P. fluorescens, other T6SS-delivered effectors continue to support substantial killing activity in the absence of both Rhs proteins. This difference might reflect distant relatives lacking conserved immunity proteins able to at least partially neutralize other effectors (since some of the cargo effectors of Db10 are conserved in other Serratia strains, e.g., Tai4/Tae4 proteins [Fig. 1A]) and/or the tailoring of effector specificities to favor non-Serratia targets. For example, Ssp1 (Tae4.1 of S. marcescens [Tae4.1^SM^]) has peptidoglycan hydrolase activity but is relatively ineffective against S. marcescens Db10 (21, 23).

Rhs2-CT was shown to be a DNase toxin, similar to RhsA and RhsB of D. dadantii (16), so far the only other T6SS-delivered Rhs toxins for which a mode of action has been verified. Although both have HNH motifs, Rhs2-CT is not closely related to RhsB-CT (only 31% identity, mostly around the HNH motif) and RhsI2 is unrelated to RhsI_B_, suggesting that Rhs2-CT/RhsI2 represent a different nuclease toxin/immunity subfamily. Rhs1-CT, on the other hand, is a distinct, novel antibacterial toxin which appears to have a cytoplasmic site of action. We also noted that the full-length Rhs1 protein is less toxic than the isolated CT alone and that, while we were unable to make a single Δ*rhsI1* mutant due to self-toxicity, a strain carrying the *rhsI* deletion in a T6SS mutant background (Δ*rhsI1* Δ*tssH* mutant) was healthy. Since we have shown previously that the Δ*tssH* deletion does not have a polar effect on other genes (7, 21), the present finding implies that self-toxicity results only from effectors injected by neighboring cells. These data are consistent with the idea that Rhs1-CT is a cytoplasmic-acting toxin but that part of the full-length Rhs protein encapsulates the toxic CT domain, sequestering it from the cytoplasm prior to secretion. The basis for this prediction comes from an important structural study showing that Rhs repeats within insecticidal toxin proteins form a shell-like structure around a toxic C-terminal domain (29). Somehow, delivery of Rhs1 by the T6SS must trigger release of the CT from this Rhs repeat shell, either before or after its entry into a target cell. While the details of this release process remain to be determined, it is likely that the CT is proteolytically cleaved from the Rhs domain. The study described above (29) also revealed that conserved residues within the Rhs hyperconserved domain form an aspartic acid protease active site for autoproteolysis of the CT domain; these critical residues are conserved in the T6SS-delivered Rhs1.

Parallels can readily be drawn between Rhs and contact-dependent growth inhibition (Cdi) systems, which also mediate interbacterial antagonism (16, 17). CdiA proteins are also large polymorphic toxins, containing N-terminal transport domains and highly variable CT toxin domains and having cognate CdiI immunity proteins encoded by genes immediately downstream of each CdiA-CT. CdiA proteins are secreted and anchored on the outside of the producing cell, where they interact with specific receptors on target cells, and the toxin domains are imported into the target cell to exert their action (17, 30, 31). The similarity between Cdi and Rhs toxins, and the fact that Rhs proteins can be detected on the cell surface (28), leads to the idea that following translocation out of the cell by the T6SS, Rhs proteins remain anchored on the outside of the producing cell, where they interact with the target cell. In comparing the *cdiB-cdiA-cdiI* genetic organization of Cdi systems, where CdiB interacts with the N-terminal domain of CdiA to mediate its translocation and surface anchoring, to the *eagR1-rhs1-rhsI1* arrangement, EagR1 becomes an obvious candidate for a surface-anchoring and/or translocation factor for Rhs1. Consistent with this idea, we have shown that EagR1 is specifically required for Rhs1-mediated toxicity; thus, we have identified a new component of T6SS-mediated toxin delivery.

EagR1 is not unique to S. marcescens Db10 but rather appears to represent a conserved accessory factor for certain T6SS-delivered toxins in many organisms. We observed that genes encoding EagR1-family proteins very often are located directly upstream of T6SS-associated *rhs* genes. Zhang et al. independently noted this association during an extensive bioinformatic study (19), suggesting that these proteins serve as an adaptor between polymorphic toxin and T6SS. In addition to this genomic evidence, the IdrC protein of P. mirabilis is an EagR1-family protein and has been reported to be essential for T6SS-mediated self-recognition, as is the Rhs family protein IdrD, encoded immediately downstream of it (27). It is important to note that not all T6SS-associated Rhs proteins are encoded adjacent to an EagR1 family protein, suggesting that there are other means of achieving the same function. For example, in Yersinia pseudotuberculosis, a tetratricopeptide repeat protein is encoded by a gene in a genetic context equivalent to that of *eagR1* and *idrC* (17) and may fulfill an equivalent function. Furthermore, EagR1-like proteins also can be associated with PAAR-containing proteins other than Rhs proteins. For example, the DUF1795 protein PA0094 is encoded by a gene immediately upstream of that encoding Tse6/PA0093 in P. aeruginosa. Tse6/PA0093 is a T6SS-delivered antibacterial effector which contains an N-terminal PAAR domain and C-terminal Toxin_61 domain (25, 26). This observation suggests that the role of EagR1 is intimately linked with the PAAR-containing domain of Rhs and with T6SS-dependent translocation/localization, rather than, for example, release of the CT from the Rhs repeat shell. This is supported by a phylogenetic tree of EagR1 family proteins (see Fig. S2B in the supplemental material), which implies that EagR1 family proteins whose associated Rhs proteins are of the same clade are most closely related to each other, and it is the N-terminal, PAAR-containing regions of Rhs proteins that determine clade (18).

Several possible roles for EagR1 consistent with our data can be envisaged. It may act as a delivery chaperone for Rhs1, perhaps binding the N-terminal PAAR-containing domain and delivering it to its binding site on VgrG during assembly of a primed secretion system. Alternatively, EagR1 might be required for anchoring of Rhs1 on the outside of the producing cell, following its translocation across the outer membrane by the T6SS. Since EagR1 does not resemble an intrinsic outer membrane protein or lipoprotein, this most likely would be via interaction with a resident outer membrane protein(s). The crystal structure of PA0094 exhibits a Mog1p/PsbP-like fold (PDB entry 1TU1). PsbP is believed to function as an assembly and stability factor within the plant photosystem II complex and Mog1 as a regulatory protein interacting with Ran GTPase in yeast (32). Therefore, EagR1 family proteins likely also participate in protein-protein interactions and perhaps regulate complex formation. An appealing idea from our structural model is that EagR1 uses its conserved concave face to clasp a partner, perhaps part of the N-terminal domain of Rhs1.

We propose that there are many other families of effector-specific accessory proteins apart from the EagR1-like proteins. For example, VasW is an unrelated protein required for delivery of the membrane-targeting VasX effector in V. cholerae (33). VasW is encoded by a gene immediately upstream of *vasX* and is required for VasX secretion, although its precise function is unknown. Consequently, we propose that Eag (effector-associated gene) be used as a new general nomenclature for accessory proteins associated with T6SS effectors. Thus, while EagR would refer to the DUF1795 Rhs-associated proteins identified here, EagV could be used for VasW, associated with VasX, and EagP for PA0094, associated with the simpler PAAR effector Tse6/PA0093. This nomenclature would parallel the Tag (for T6S-associated gene) nomenclature for accessory secretion system components, such as TagF (34).

In conclusion, we have shown that Rhs effectors delivered by the T6SS can represent primary determinants of intraspecies competition as well as important contributors to interspecies competition in Gram-negative bacteria. EagR has been identified as a conserved accessory factor essential for the successful deployment of a specific Rhs effector by the T6SS, and many distinct families of such Eag proteins may exist to assist different classes of T6SS-delivered effectors. The varied effector portfolio of the T6SS will be central to the ability of this system to play a key role in shaping diverse interbacterial interactions and bacterial communities.

## Supplementary Material

Supplemental material
