# Supplementary material for: Intraspecies Competition in Serratia marcescens Is Mediated by Type VI-Secreted Rhs Effectors and a Conserved Effector-Associated Accessory Protein
Source: J Bacteriol. 2015 Jun 19;197(14):2350–60. doi: 10.1128/JB.00199-15 (PMC4524185; doi:10.1128/JB.00199-15)
Supplement: Supplemental material [file supp_197_14_2350__index.html]

Supplemental material 

# Intraspecies Competition in Serratia marcescens Is Mediated by Type VI-Secreted Rhs Effectors and a Conserved Effector-Associated Accessory Protein

## Supplemental material

- Supplemental file 1 -

  Fig. S1 (Putative partial HNH endonuclease domain), S2 (Sequence-based comparison of EagR1 with other DUF1795 family proteins), and S3 (Structure model of EagR1) and Table S1 (Primer sequences and plasmid construction)

  PDF, 2.5M
