# Supplementary material for: Intraspecies Competition in Serratia marcescens Is Mediated by Type VI-Secreted Rhs Effectors and a Conserved Effector-Associated Accessory Protein
Source: J Bacteriol. 2015 Jun 19;197(14):2350–60. doi: 10.1128/JB.00199-15 (PMC4524185; doi:10.1128/JB.00199-15)
Supplement: Supplemental material [file JB.00199-15_zjb999093663so1.pdf]

Supplemental Information

Intra-species Competition in *Serratia marcescens* is Mediated by Type VI-Secreted Rhs Effectors and a Conserved Effector-Associated Accessory Protein

Juliana Alcoforado Diniz and Sarah J. Coulthurst

FIGURE S1

A

Sequence of Rhs2-CT (SMDB11_1610, amino acids 1290-1430):

```
SNCSTLDRIIGDANKVASRGGAITAKQAQILRDNLPVVQRRSVFQNQMARKEFV
RDQHYLMSQWEANTGRTWPTGATPHHIIPLESGGANKWWNLMPTHGTLPNHSLP
GVPGPHAAGGVLRTTVQQSRKALPPGTITDLRL
```

B

Conserved domain: cd00085 (HNH nucleases)

Conserved domain length: 57 Bit Score: 36.30 E-value: 1.66e-04

```
                        10        20
                ....*....|....*....|.
Rhs2-CT       79 HHIIPLESGGANKWWNLMPTH 99
cd00085       29 DHIIPLSDGGNNDLDNLVLLC 49
```

Conserved domain: pfam01844 (HNH endonuclease)

Conserved domain length: 47 Bit Score: 35.35 E-value: 3.78e-04

```
                          10        20
                  ....*....|....*....|.
Rhs2-CT         79 HHIIPLESGGANKWWNLMPTH 99
pfam01844       17 DHIIPRSKGGADDLSNLVLLC 37
```

FIG S1. The putative partial HNH endonuclease domain in the C-terminal domain of *Serratia marcescens* Db10 Rhs2 (Rhs2-CT). (A) The amino acid sequence of Rhs2-CT with the region aligning with part of the HNH conserved domain underlined and candidate residues making up the HNH motif in red. (B) Sequence alignments of conserved domains cd00085 and pfam01844 (HNH endonuclease) with Rhs2-CT, generated using the NCBI Conserved Domain Search through the BLAST interface (http://www.ncbi.nlm.nih.gov/).

FIGURE S2

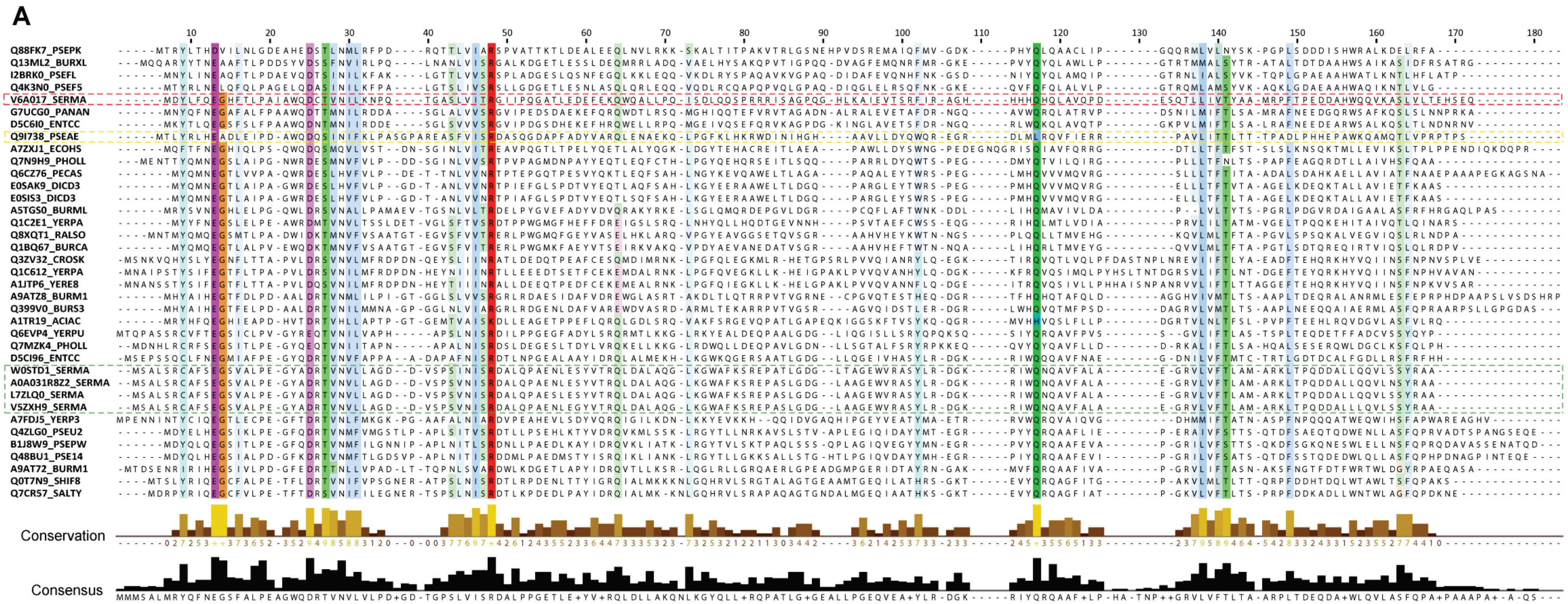

Continued...

B

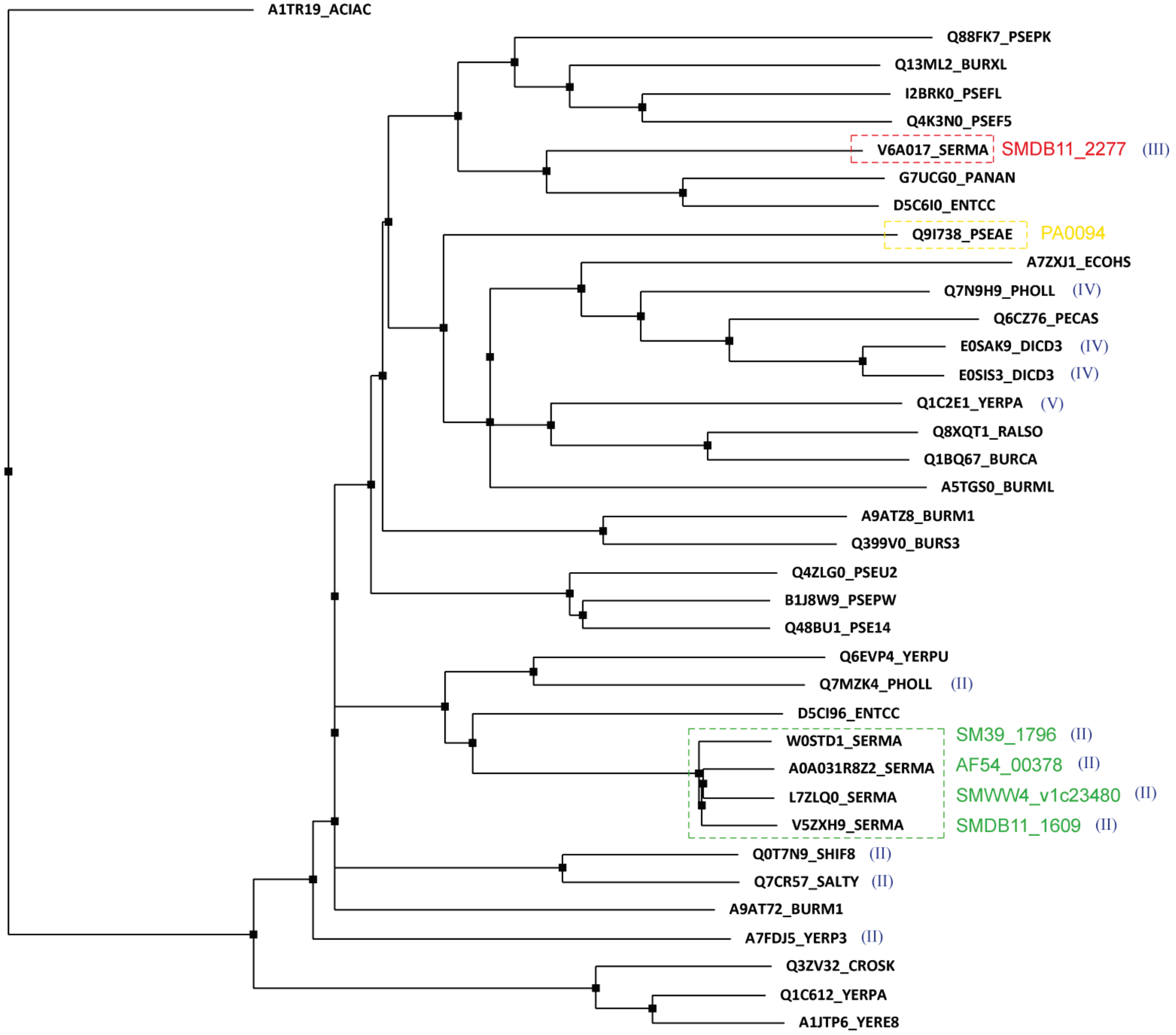

C

Entry	Entry name	Gene name	Organism
A0A031R8Z2	A0A031R8Z2_SERMA	AF54_00378	*Serratia marcescens* BIDMC 81
A1JTP6	A1JTP6_YERE8	YE2704	*Yersinia enterocolitica* NCTC 13174
A1TR19	A1TR19_ACIAC	Aave_2839	*Acidovorax citrulli* AAC00-1
A5TGS0	A5TGS0_BURML	BMA721280_L0139	*Burkholderia mallei* 2002721280
A7FDJ5	A7FDJ5_YERP3	YpsIP31758_0329	*Yersinia pseudotuberculosis* IP 31758
A7ZXJ1	A7ZXJ1_ECOHS	EcHS_A0615	*Escherichia coli* O9:H4 (strain HS)
A9AT72	A9AT72_BURM1	BMULJ_05444	*Burkholderia multivorans* ATCC 17616
A9ATZ8	A9ATZ8_BURM1	BMULJ_06208	*Burkholderia multivorans* ATCC 17616
B1J8W9	B1J8W9_PSEPW	PputW619_2519	*Pseudomonas putida* W619
D5C6I0	D5C6I0_ENTCC	ECL_01566	*Enterobacter cloacae* ATCC 13047
D5CI96	D5CI96_ENTCC	ECL_03141	*Enterobacter cloacae* ATCC 13047
E0SAK9	E0SAK9_DICD3	Dda3937_01759	*Dickeya dadantii* 3937
E0SIS3	E0SIS3_DICD3	Dda3937_02772	*Dickeya dadantii* 3937
G7UCG0	G7UCG0_PANAN	PAGR_g1688	*Pantoea ananatis* PA13
I2BRK0	I2BRK0_PSEFL	PflA506_5309	*Pseudomonas fluorescens* A506
L7ZLQ0	L7ZLQ0_SERMA	SMWW4_v1c23480	*Serratia marcescens* WW4
Q0T7N9	Q0T7N9_SHIF8	SFV_0313	*Shigella flexneri* 5b 8401
Q13ML2	Q13ML2_BURXL	Bxe_B1282	*Burkholderia xenovorans* LB400
Q1BQ67	Q1BQ67_BURCA	Bcen_3343	*Burkholderia cenocepacia* AU 1054

Entry	Entry name	Gene name	Organism
Q1C2E1	Q1C2E1_YERPA	YPA_3419	*Yersinia pestis* bv. Antiqua
Q1C612	Q1C612_YERPA	YPA_2145	*Yersinia pestis* bv. Antiqua
Q399V0	Q399V0_BURS3	Bcep18194_B0647	*Burkholderia cepacia* ATCC 17760
Q3ZV32	Q3ZV32_CROSK		*Cronobacter sakazakii*
Q48BU1	Q48BU1_PSE14	PSPPH_5071	*Pseudomonas syringae pv phaseolicola* 1448A
Q4K3N0	Q4K3N0_PSEF5	PFL_6095	*Pseudomonas fluorescens* Pf-5
Q4ZLG0	Q4ZLG0_PSEU2	Psyr_4985	*Pseudomonas syringae* pv. *syringae* B728a
Q6CZ76	Q6CZ76_PECAS	ECA4277	*Pectobacterium atrosepticum* SCRI 1043
Q6EVP4	Q6EVP4_YERPU	api90	*Yersinia pseudotuberculosis*
Q7CR57	Q7CR57_SALTY	STM0290	*Salmonella typhimurium* LT2
Q7MZK4	Q7MZK4_PHOLL	plu4281	*Photorhabdus luminescens subsp laumondii* TT01
Q7N9H9	Q7N9H9_PHOLL	plu0354	*Photorhabdus luminescens subsp laumondii* TT01
Q88FK7	Q88FK7_PSEPK	PP_4084	*Pseudomonas putida* KT2440
Q8XQT1	Q8XQT1_RALSO	RSp1138	*Ralstonia solanacearum* GMI1000
Q9I738	Q9I738_PSEAE	PA0094	*Pseudomonas aeruginosa* PAO1
V5ZXH9	V5ZXH9_SERMA	SMDB11_1609	*Serratia marcescens* Db11
V6A017	V6A017_SERMA	SMDB11_2277	*Serratia marcescens* Db11
W0STD1	W0STD1_SERMA	SM39_1796	*Serratia marcescens* SM39

Continued...

FIG S2. Sequence-based comparison of EagR1 with other DUF1795 family proteins.

(A) Multiple sequence alignment of EagR1 (SMDB11_2277; boxed in red) with other members of the DUF1795 (PF08786) family, including homologues in *S. marcescens* (boxed in green), PA0094 from *P. aeruginosa* (boxed in yellow) and homologues associated with previously-described Rhs family proteins (see text). Alignment was generated using Clustal Omega and visualised using Jalview. The alignment is coloured by conservation according to the ClustalX colour scheme and the conserved residues (coloured; conservation score ≥ 7) are those highlighted in Fig. S3. Proteins are labelled by UniProt entry name. (B) Neighbour-joining tree calculated from the multiple sequence alignment in Part A. Homologues of particular interest are highlighted by genomic identifier. Roman numerals in parentheses refer to the clade of the associated Rhs protein, when this is one of the Enterobacterial Rhs proteins included in the analysis of A. P. Jackson, G. H. Thomas, J. Parkhill and N. R. Thomson (BMC Genomics, **10**:584, 2009). (C) Table summarising the details of each UniProt entry for parts A and B.

FIGURE S3

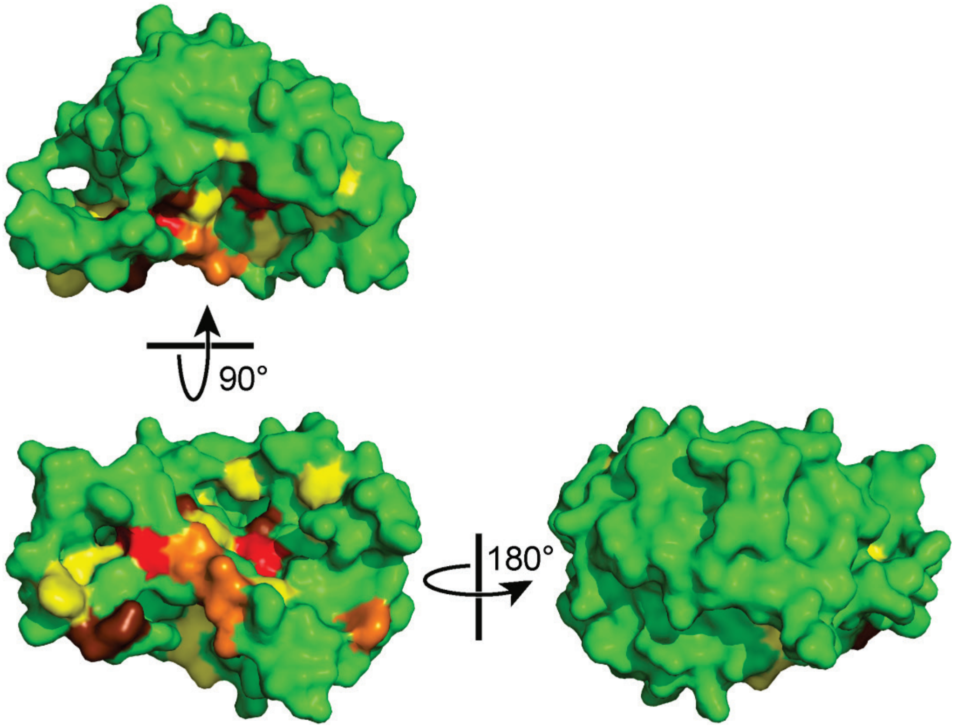

FIG S3. Structure model of EagR1 from *S. marcescens* Db10 generated using the DUF1795 family protein PA0094 (PDB 1TU1).

Based on the multiple sequence alignment in Fig. S2, residues conserved in the DUF1795 family are coloured from brown (highest conservation, score 10) through red and orange to yellow (less conserved, score 7); poorly or non-conserved residues are shown in green.

TABLE S1. Primer sequences and details of plasmid construction

Plasmid	Sequence of relevant primers (5'-3')[1]	Description of primers or other cloning details
pSC635	TATATCTAGAAGAGGAATACATATGAAGCCACGATGCGCAGC	Forward primer to clone SMDB11_2278 incorporating RBS[2] into pBAD18-Kan (*Xba*I)
	TATAGCATGCCATTTTACAATCCAATTTCAAAAGGC	Reverse primer to clone SMDB11_2278 into pBAD18-Kan (*Sph*I)
pSC636	TGTGGAATTCAGAGGATTGTAAAATGCAACTAGATACTTATGACG	Forward primer to clone SMDB11_2278A into pBAD18-Kan (*Eco*RI)
	TGTGGAGCTCTTAATAAATTATGTTATTCCTTTTGCAATATTTTCG	Reverse primer to clone SMDB11_2278A into pBAD18-Kan (*Sac*I)
pSC637	TATATCTAGAAGAGGAATACATATGAAGCCACGATGCGCAGC	Forward primer to clone SMDB11_2278 incorporating RBS into pBAD18-Kan (*Xba*I)
	TGTGGCATGCTTAATAAATTATGTTATTCCTTTTGCAATATTTTCG	Reverse primer to clone SMDB11_2278+SMDB11_2278A into pBAD18-Kan (*Sph*I)
pSC640	n/a	Synthetic insert (Invitrogen) encoding full length SMDB11_2278A fused with an HA tag (YPYDVPDYA) immediately at its C-terminus, and with an RBS-containing sequence (CAGAGGATTGTAAA) 5' of the start codon, all cloned into pBAD18-Kan (*Eco*RI/*Sac*I)
pSC643	TATAGAGCTCAGAGGAATACATATGGCTGAGATCTGGGCAGC	Forward primer to amplify first sector of SMDB11_2278 incorporating new RBS and *Nde*I site (*Sac*I)
	CCATAATCCCAGAAGCTTTGCG	Reverse primer to amplify first sector of SMDB11_2278 (*Hin*dIII)
	TCGCAAAGCTTCTGGGATTATGG	Forward primer to amplify second sector of SMDB11_2278 (*Hin*dIII)
	CAGGTAGCGCTGGGTATGG	Reverse primer to amplify second sector of SMDB11_2278 (*Cla*I)
	TACGAGATCGATGCGCTCGG	Forward primer to amplify third sector of SMDB11_2278 (*Cla*I)
	TAATCTCGAGTCTAGACATTTTACAATCCAATTTCAAAAGGC	Reverse primer to amplify third sector of SMDB11_2278 (*Xho*I)
pSC658	TATATCTAGATGATTACTTGCAAGGACATCC	Forward primer to clone SMDB11_2277 incorporating RBS into pBAD18-Kan (*Xba*I)
	TATAGCATGCCTATTGTTCACTGTGTTCGGTCAAGAC	Reverse primer to clone SMDB11_2277 into pBAD18-Kan (*Sph*I)
pSC672	TATAGAGCTCAGAGGATGAGATTGTGACATGAATGAATTTGAC	Forward primer to clone SMDB11_1611 incorporating RBS into pBAD18-Kan (*Sac*I)
	TGTGGCATGCTTAAATGGTAACTATCTTATCTTTTGCTATACCC	Reverse primer to clone SMDB11_1611 into pBAD18-Kan (*Sph*I)
pSC673	n/a	Insert digested from pSC672 and cloned into pSC636 (*Sac*I/*Sph*I)
pSC674	TATACCATGGATGGGCAGTAATTGTTCGACTCTTGACCG	Forward primer to clone SMDB11_1610 into pET15b-TEV (*Nco*I)
	TGTGCTCGAGTCACAATCTCAAATCAGTTATAGTTCCAG	Reverse primer to clone SMDB11_1610 into pET15b-TEV (*Xho*I)
pSC675	TATACCATGGATGGGCAGTAATTGTTCGACTCTTGACCG	Forward primer to clone SMDB11_1610 into pET15b-TEV (*Nco*I)
	TGTGCTCGAGTTAAATGGTAACTATCTTATCTTTTGCTATACCC	Reverse primer to clone SMDB11_1610+SMDB11_1611 into pET15b-TEV (*Xho*I)
pSC619	TATGACTAGTCTGGATTACTCCCAAAACCTGC	Forward primer to clone upstream flanking region of SMDB11_2278A for marker exchange (*Spe*I)
	TGTGAAGCTTTAGTTGCATTTTACAATCCAATTTCAAAAGG	Reverse primer to clone upstream flanking region of SMDB11_2278A for marker exchange (*Hin*dIII)
	GCGCAAGCTTATAATTTATTAAAAAAGAGGAAACTCATCCAC	Forward primer to clone downstream flanking region of SMDB11_2278A for marker exchange (*Hin*dIII)
	TATAGGGCCCTCAGAGTGTTATAAAGATTTCAGTGG	Reverse primer to clone downstream flanking region of SMDB11_2278A for marker exchange (*Apa*I)

pSC649	TATATCTAGACGTCTATAACGAAGCCAGCATG	Forward primer to clone upstream flanking region of SMDB11_2277 for marker exchange (*Xba*I)
	TATAGTCGACGTAATCCATGGATGTCCTTGCAAG	Reverse primer to clone upstream flanking region of SMDB11_2277 for marker exchange (*Sal*I)
	TATAGTCGACGAACACAGTGAACAATAGCCATGG	Forward primer to clone downstream flanking region of SMDB11_2277 for marker exchange (*Sal*I)
	TATAGGGCCCGTTGAACGGTCATTGCTGC	Reverse primer to clone downstream flanking region of SMDB11_2277 for marker exchange (*Apa*I)
pSC650	TATAACTAGTTCTGTATATGACGGTTTGATTCCC	Forward primer to clone upstream flanking region of SMDB11_1610 for marker exchange (*Spe*I)
	TATAAAGCTTCGCTTCGGTCATCGTAAGGTC	Reverse primer to clone upstream flanking region of SMDB11_1610 for marker exchange (*Hin*dIII)
	TATAAAGCTTACTGATTTGAGATTGTGACATGAATG	Forward primer to clone downstream flanking region of SMDB11_1610 for marker exchange (*Hin*dIII)
	TATAGGGCCCGTGTGATACCCGCATATATTCGC	Reverse primer to clone downstream flanking region of SMDB11_1610 for marker exchange (*Apa*I)
pSC664	TATAACTAGTTCTGTATATGACGGTTTGATTCCC	Forward primer to clone upstream flanking region of SMDB11_1610 for marker exchange (*Spe*I)
	TATAAAGCTTCGCTTCGGTCATCGTAAGGTC	Reverse primer to clone upstream flanking region of SMDB11_1610 for marker exchange (*Hin*dIII)
	TATAAAGCTTGATAAGATAGTTACCATTTAATATTTGTTGACTGC	Forward primer to clone downstream flanking region of SMDB11_1611 for marker exchange (*Hin*dIII)
	TATAGGGCCCTCTTCTAAATCTTACTTCGTAATTGCC	Reverse primer to clone downstream flanking region of SMDB11_1611 for marker exchange (*Apa*I)
pSC827	TATATCTAGAGGATCTCAACCAGCCTGGC	Forward primer to clone upstream flanking region of SMDB11_2278 for marker exchange (*Xba*I)
	ATATAAGCTTCTCAGCCATGGCTATTGTTCAC	Reverse primer to clone upstream flanking region of SMDB11_2278 for marker exchange (*Hin*dIII)
	TATAAAGCTTATTGATAAGCCTTTTGAAATTGGATTG	Forward primer to clone downstream flanking region of SMDB11_2278 for marker exchange (*Hin*dIII)
	TATAGGGCCCACACACACTCTGCATTATGGAATG	Reverse primer to clone downstream flanking region of SMDB11_2278 for marker exchange (*Apa*I)

[1]Restriction sites underlined

[2]RBS, ribosome binding site
